# *Dictyosteliumdiscoideum–Mycobacterium marinum* infection model: a powerful high-throughput screening platform for anti-infective compounds

**DOI:** 10.3389/fmicb.2025.1612354

**Published:** 2025-07-04

**Authors:** Jahn Nitschke, Nabil Hanna, Thierry Soldati

**Affiliations:** Department of Biochemistry, Faculty of Science, University of Geneva, Geneva, Switzerland

**Keywords:** anti-infectives, phenotypic screening, *Mycobacterium marinum*, *Dictyostelium discoideum*, high-throughput

## Abstract

Tuberculosis is among the world’s deadliest diseases, causing approximately 2 million deaths annually. The urgent need for new antitubercular drugs has been intensified by the rise of drug-resistant strains. Despite recent advancements, most hits identified through traditional target-based screening approaches exhibit limited efficacy *in vivo*. Consequently, there is a growing demand for whole-cell-based approaches that utilize host-pathogen systems directly. The *Dictyostelium discoideum*–*Mycobacterium marinum* host–pathogen system is a well-established and powerful alternative model system for studying mycobacterial infections. In this article, we present a phenotypic host–pathogen assay protocol that monitors *M. marinum* during infection of the amoeba *D. discoideum*. This assay is characterized by its scalability for high-throughput screening, robustness, and ease of manipulation, making it an effective system for compound screening. Notably, this system provides dual readouts: bacterial load via a bioluminescent *M. marinum* strain and host survival and growth via a fluorescent *D. discoideum* strain, enabling further host characterization by quantifying growth inhibition and potential cytotoxicity. Finally, the assay was benchmarked against selected antibiotics and anti-infectives, and IC_50_s and MIC values were calculated where applicable, demonstrating its ability to differentiate between antibiotics and anti-infective compounds.

## Introduction

*Dictyostelium discoideum* (Dd) is a social amoeba and a professional phagocyte. As such, it shares conserved and fundamental cell-autonomous immunity mechanisms with innate immune phagocytes of animals. Additionally, it is genetically tractable, has a fully sequenced genome (Eichinger et al., 2005), and is readily infected by a range of intracellular pathogens, such as *Legionella pneumophila* (Solomon and Isberg, 2000; Welin et al., 2023), as well as *Vibrio cholerae*, *Francisella noatunensis*, *Pseudomonas aeruginosa*, *Salmonella enterica,* and *Mycobacterium* species, as previously reviewed (Dunn et al., 2018). Mycobacteria are a genus of bacteria characterized by their waxy cell walls (Batt et al., 2020). Several strains are pathogenic to humans and animals, most notably members of the *Mycobacterium tuberculosis* (Mtb) complex (Kanabalan et al., 2021). A close relative of this complex is *Mycobacterium marinum* (Mm) (Stinear et al., 2008), a facultative human pathogen that can cause skin lesions similar to the lung lesions observed in active tuberculosis (TB) (Ramakrishnan, 2004; Tobin and Ramakrishnan, 2008). Mm serves as a convenient substitute for Mtb, as it retains key virulence strategies but has a shorter doubling time (approximately 8 h) and can be handled under biosafety level 2 (BSL-2) conditions, unlike Mtb, which requires BSL-3 infrastructure (Ramakrishnan, 2004). Historically, TB drug discovery has relied on screening attenuated strains, such as the *Mycobacterium bovis* Bacillus Calmette–Guérin strain (Taneja and Tyagi, 2007), which is also used as a live vaccine (Calmette et al., 1927), or non-pathogenic strains, such as *Mycobacterium smegmatis (*Altaf et al., 2010; Andries et al., 2005). While this screening strategy is resource-efficient, it is associated with a high attrition rate, largely due to its limited reflection of the complex biology underlying mycobacterial infections. As a more biologically relevant alternative, screening directly on infected host cells, such as murine Raw264.7 macrophages or human THP-1 macrophages, has become an accepted strategy (Christophe et al., 2010; Pethe et al., 2013; Schaaf et al., 2016). This approach not only incorporates aspects of infection biology and pharmacokinetics but also opens opportunities to identify anti-infective compounds that act on the host, either exclusively or in combination (Tobin, 2015; Udinia et al., 2023). However, a major bottleneck in phenotypic screens is determining the compound’s mode of action, which is particularly challenging when using resource-intensive systems, such as Mtb or human primary macrophages, which are difficult to maintain and genetically manipulate. To bridge this gap, we propose the Dd–Mm infection model. We have recently demonstrated the utility of this model for investigating host–pathogen interactions (López-Jiménez et al., 2018; Raykov et al., 2023) and for identifying active anti-infective compounds (Hanna et al., 2020; Kirchhoffer et al., 2023; Kirchhoffer et al., 2024; Nitschke et al., 2024; Trofimov et al., 2018). Consequently, we have since developed this system into a high-throughput platform capable of monitoring not only bacterial load via a bioluminescent Mm strain (Andreu et al., 2010; Arafah et al., 2013) but also host survival and growth via a fluorescent Dd strain.

Additionally, both wild-type strains can be readily substituted with genetically modified mutants, allowing researchers to determine whether a compound’s target lies within the host or the pathogen. This feature enables further applications, including genome-wide functional screens, such as REMI-seq in Dd (Kuspa, 2006), Tn-seq in Mm (Lefrançois et al., 2024), or dual RNA-seq across both organisms (Hanna et al., 2019). We are convinced that this workflow will enable the detailed elucidation of cell-autonomous defense mechanisms as well as virulence strategies, providing the resolution needed to either enhance or inhibit them and ultimately contributing to the development of new therapies for mycobacterial infections. This methods and protocols article builds on a recent methods article that illustrated the experimental versatility of Dd with a focus on microscopy-based techniques (Mottet et al., 2021) and aims to provide detailed technical guidance on using the high-throughput Dd–Mm platform to screen chemical and natural compounds for antibacterial and anti-infective activities. In parallel, the generated data analysis workflow represents a powerful and adaptable tool for evaluating anti-infective compounds in high-throughput assays.

## Materials and methods

### Cell culture

- *D. discoideum* laboratory strain Ax2(Ka) [20] (the strains can be obtained from the stock center, http://www.dictybase.org/StockCenter/StockCenter.html, ID: DBS0235521).
- *D. discoideum* laboratory strain Ax2(Ka), transformed with Cherry-Act5, resistant to hygromycin.
- HL5c medium, including glucose, supplemented with vitamins and microelements (ForMedium): Resuspend 26.55 g of powder in 1 L of deionized water. Filter-sterilize (Steritop 0.22 μm filters, Millipore).
- Penicillin/streptomycin 100 × stock solution: 10,000 U/ml and 10,000 μg/ml, respectively.
- Hygromycin: Prepare a 1,000 × stock solution by dissolving 100 mg of powder in 1 ml of deionized water. Filter-sterilize and store at −20°C.
- 10 cm cell culture dishes made of tissue culture-treated polystyrene.

### Mycobacterial culture

- *M. marinum* M strain variants expressing Lux, or GFP (e.g., pmsp12: GFP); kanamycin-resistant.
- 7H9 medium (Difco): dissolve 4.7 g of Middlebrook 7H9 powder in 900 ml of deionized water. Add 500 μl of Tween 80 and 2 ml of glycerol, then autoclave. Allow the medium to cool, and then add 10% v/v of Middlebrook OADC supplement (Becton Dickinson). Filter-sterilize and store at 4°C.
- Kanamycin: Prepare a 1,000 × stock solution by dissolving 30 mg of the powder in 1 ml of deionized water. Filter-sterilize the solution and store it at −20°C.
- Rifabutin: Prepare a 1,000 × stock solution by dissolving 10 mg of powder in 1 ml of deionized water. Filter-sterilize and store at −20°C.
- Glass beads, 5 mm diameter.

## Protocol

### Preparation of media and cell culture material

#### Mycobacterium marinum

##### 7H9 for liquid cultures

- Use a measuring cylinder to dissolve 2.35 g of 7H9 powder (BD, Difco Middlebrook 7H9) in 450 ml of double-distilled water.
- Add 1 ml of glycerol (Sigma Aldrich CAS-No: 56-81-5, suitable for cell culture, final concentration 0.2%).
- Add 250 μl of Tyloxapol (Merck T8761-50G, final concentration 0.05%). To reduce the viscosity of Tyloxapol and facilitate pipetting, briefly heat it in a microwave for easier handling.
- Stir the mixture until it is clear.
- Filter-sterilize (Steritop, 0.22 μm PES) in a laminar flow hood, then add 50 ml of OADC (BD BBL, Middlebrook Oleic Albumin Dextrose Catalase Growth Supplement), with aliquots filtered and sterilized in advance and stored at 4°C.
- Store medium at 4°C until ready for use. Prewarming is not necessary.

##### 7H11 for agar plates

- Dissolve 11.67 g of 7H11 Agar (BD, Difco 7H11) in 500 ml of double-distilled water directly in a 500 ml bottle.
- Add 2.5 ml of glycerol (final concentration 0.5%), mix thoroughly by shaking, and autoclave. Autoclaving will completely dissolve the agar.
- Use immediately after autoclaving or reheat later in the microwave. When the agar is liquid after autoclaving or microwaving, take the necessary volume of agar to the plate.
- Add OADC (10% of the total volume).
- Add the appropriate antibiotic selection (kanamycin at 50 μg/ml, hygromycin at 100 μg/ml, or rifabutin at 10 μg/ml final concentration) and plate approximately 20 ml into a 10 cm Petri dish. Leave the Petri dishes standing for at least 30 min under the hood to cool and dry.
- Stored at 4°C, plates can be used for up to 1 month.

#### Dictyostelium discoideum

##### Filter-sterilized HL5-C medium

- To prepare five bottles of 900 ml each, dissolve 119.25 g of HL5-C powder (Formedium, stored at 4°C) in 4.5 L of double-distilled water. Shake the powder bottle before use to ensure a homogeneous medium preparation.
- Stir for at least 1 h until fully dissolved.
- Check the pH using pH paper; it should be 6.5.
- Filter-sterilize under a laminar flow hood into 1 L Schott bottles and store at 4°C for up to 3 months. Then, use as needed. Prewarming to room temperature is recommended. After opening a bottle for the first time, store it at room temperature.

##### Antibiotics in the culture medium

Alternatively, penicillin/streptomycin (P/S; 10 mg/ml and 10,000 U/ml, respectively; aliquots stored at −20°C) can be added after the first bottle is used up. The final concentrations in the medium should be 100 μg/ml and 100 U/ml. While this helps prevent contamination, it can make it more difficult to detect resistant strains. Therefore, the addition of P/S is not recommended for maintenance culture.

##### Fluorescence of HL5-C in GFP channel

HL5-C has a significant autofluorescence background at GFP emission wavelengths. This is problematic since it makes segmentation of images acquired at the high-content microscope difficult and adds an offset signal to readouts acquired with a plate reader. This background signal could not be attributed to a specific ingredient in the medium, but storage at room temperature under constant lighting reduced its intensity.

### Maintenance of cell and bacterial cultures

#### Mycobacterium marinum

##### Cycle of plate culture, primary culture, and liquid stocks

A fresh culture of Mm LuxCDABE (Andreu et al., 2010) or Mm pMSP12: GFP (Addgene plasmid # 30167) (Davis et al., 2002) (derived from *M. marinum* M strain ATCC BAA-535) is plated every 3 months. Starter cultures and liquid stocks from a plate culture should be used for a maximum of 1 month before preparing a new starter culture. Generally, passage numbers should be kept low for mycobacteria culture, and it is recommended to use a primary liquid culture as an inoculum for each infection experiment.

##### Plate culture and primary liquid culture

- Recover Mm from a - 80°C glycerol stock and start a plate culture using an inoculation loop. Then, streak the culture onto a 7H10 agar plate.
- After sealing the plates with parafilm, incubate them at 32°C in a box to maintain humidity by adding a wet paper tissue. After 3 days, growth should be visible, depending on the inoculum size. After approximately 7 days, the colonies should have grown sufficiently to store the plates at 15°C.
- Start a primary liquid culture (10–20 ml) using the 7H10 plate culture in an Erlenmeyer flask with a gas-permeable plug.
- Syringe the inoculum 10 times using a blunt needle (25 Gx3/4″ blunt needles, preferably 3 ml syringes; aspirating and releasing once counts as one syringed suspension). Make sure to wear a needle-proof glove to prevent injury.
- Prepare the Erlenmeyer flask with 7H9 medium and the appropriate antibiotic selection (see Supplementary Table 3).
- Add the inoculum from the syringe to the Erlenmeyer flask.
- Incubate the culture at 32°C while shaking at 150 rpm for a minimum of 24 h.
- This culture can now be used to inoculate liquid cultures for infection experiments, while the remaining cultures can be stored in the refrigerator at 15°C. Liquid stocks should be used for a maximum of 1 month.

###### No glass beads in liquid cultures

Note: Glass beads with a diameter of 3 mm can be used in liquid cultures to limit clumping by increasing shear stress. However, alternative methods—such as using Tyloxapol instead of Tween 80 or applying short centrifugation at 20 g for 1 min—appear to be sufficient to eliminate Mm clumps.

###### Measuring OD with the Ledetect 96

Use the plate reader to measure the optical density (OD) in transparent 96-well plates. Use 200 μl of bacterial suspension per well, ensuring that no bubbles disrupt the light path in the well. Measure the OD at 620 nm and multiply the result by 2 to obtain the path length-corrected OD. Although 600 nm is the traditional wavelength for measuring OD, the machine’s closest wavelength is 620 nm. The difference in optical density (OD) at 600 and 620 nm was examined using a photometer and found to be negligible. The pathlength correction extrapolates the OD obtained across a 96-well plate well to the pathlength of a photometer cuvette (1 cm).

#### Dictyostelium discoideum

- Recover Dd Ax2(Ka) expressing mCherry from frozen spore stocks (SoerensenMC with 10% glycerol, see Supplementary Table 3) stored in liquid nitrogen. A culture should not be used for infection until it has been passaged for 1 week and is showing normal doubling times. Infection efficiency decreases with the age of a culture. After 1 month, a fresh culture must be started as described above. It is recommended to keep a record of the passage number of the current culture.
- Dd Ax2(Ka) maintenance culture should be performed following a standard protocol (Bloomfield et al., 2008; Fey et al., 2007). Briefly, cells are cultivated under adherent conditions in HL5-C medium at 22°C with constant light until reaching a maximum density of 5*10^7^ cells/10 cm Petri dish, as overgrowth has been observed to result in low infection efficiency.
- We used a splitting regime as follows: Monday (1/10 dilution), Wednesday (1/10 dilution), and Friday (1/20–1/50 dilution).

##### Measuring cell density with the countess

Countess II FL (Life Technologies, slides by Invitrogen) is used to assess cell density. After pipetting 10 μl of cell suspension into the measurement slide, incubate for 1 min to allow the cells to sediment in the focal plane before measuring. This is achieved by inserting the slide and selecting only the brightfield channel. The resulting cell count must be divided by 2 since the device assumes a 1:2 dilution in trypan blue and automatically corrects for it. Take at least two measurements to exclude measurement fluctuations. If necessary, adjust the focus manually. The practical measuring range has been reported to extend to approximately 6*10^7^ cells/ml.

### Preparations for the infection

#### Mycobacterium marinum

- From the primary liquid culture, inoculate a new liquid culture for infection. This culture should be started approximately 24 h before infection.
- For the infection culture, use an Erlenmeyer flask fitted with a gas-permeable plug. After adding an appropriate volume of 7H9 (between 10 and 30 ml, corresponding to the final culture volume) and the appropriate antibiotic, prepare the inoculum from the stored primary liquid culture.
- Resuspend by vortexing, and then pellet clumps by spinning at 300 rpm (corresponds to 20 g with the Thermo Scientific™ 75003180 rotor) for 1 min.
- Take 2–3 ml of the supernatant using a syringe (as described above) before adding the inoculum to the Erlenmeyer flask.
- Incubate the culture at 32°C while shaking at 150 rpm. Measure OD (as described above) to ensure adequate growth for the following day. If the culture is prepared 24 h before the experiment, a pathlength-corrected OD of approximately 0.3 is typically sufficient to obtain an OD of 1 on the day of the experiment.

#### Dictyostelium discoideum

- Plate Dd mCherry cells 24 h prior to the experiment in 10 cm Petri dishes at 1*10^7^ cells per Petri dish (approximately 40% confluency) in filter-sterilized HL5-C medium without penicillin/streptomycin or additional selection to prevent residual antibiotics from compromising bacterial virulence during infection.
- Incubate for 24 h under standard growth conditions at 22°C. The plates should contain approximately 3–4*10^6^ cells per ml (3–4*10^7^ cells per Petri dish), which corresponds to 90–95% confluency on the day of infection. The adherent cell lawn will be infected via spinoculation. Therefore, it should be as dense as possible while minimizing the number of floating cells.

### Infection

- Decant the Mm culture from the Erlenmeyer flask into a 50 ml Falcon conical tube and centrifuge at 20 g for 1 min, as described before.
- Aspirate the supernatant and measure the OD (as described previously). From the OD, calculate the bacterial density (see the calibration curve of OD to bacteria in Supplementary Figure 1 and Supplementary Table), and transfer the volume that corresponds to 8.75*10^8^ bacteria (MOI of 25, assuming 3.5*10^7^ Dd per Petri dish) to a fresh conical tube. During the subsequent steps, material loss might happen. Consequently, it is recommended to include an excess of 20%, for example, 1.2 * 8.75*10^8^ bacteria.
- Centrifuge the mycobacteria at 2,700 g for 10 min (equivalent to 3,500 rpm with the Thermo Scientific 75003180 rotor). After centrifugation, decant the supernatant and carefully pipette any remaining 7H9 broth.
- During the centrifugation step, aspirate the medium from the Dd culture and add 5 ml of filter-sterilized HL5-C medium without penicillin, streptomycin, or selection.
- Resuspend 600 μl (500 μl plus a 20% margin = 600 μl) of HL5-C per Petri dish to be infected.
- Syringe the suspension 10 times through a blunt needle to break up clumps, as described above, for the preparation of the infection culture. The suspension should be syringed a maximum of 10 times to avoid damage to the bacteria. Bubbles should also be avoided. It is good practice to visually check the suspension under the microscope to ensure the effectiveness of syringing.
- Add 500 μl of bacterial suspension to each Petri dish.
- Gently shake the Petri dish from side to side. Then, seal it with parafilm.
- Centrifuge twice at 500 g for 10 min (corresponds to 1,500 rpm with the Thermo Scientific™ 75003180 rotor) to accelerate sedimentation of bacteria and, consequently, enhance contact between Dd and Mm. Rotate the bucket by 180° between spins to redistribute the bacterial suspension. Shake the Petri dishes in the bucket crosswise between the first and second spins.
- Incubate for 10–20 min to allow phagocytosis. This time is not critical, but it should remain coherent throughout the experiments. In the case of a timeline experiment, the start of the phagocytosis step is marked as 0 hpi (hours post-infection).
- Wash the extracellular bacteria off with 7–10 ml of HL5-C several times (3–8 repeats, depending on the subsequent experiment). For instance, five repeats are recommended for a plate reader experiment. The washes should leave as few extracellular bacteria as possible to minimize false-positive signals. All Petri dishes should be treated equally. Special attention should be paid to adding and removing the medium at the edge of the Petri dish to avoid detaching Dd cells.
- After washing, cells are mechanically detached and resuspended in HL5-C containing fresh 5 μg/ml P/S and 5 U/ml P/S (a 1:2,000 dilution of a 10,000 μg/ml stock or 25 μl of P/S in 50 ml of HL5-C; see note on use of P/S). They are then stored in a conical tube for further use.

#### Multiplicity of infection (MOI)

The MOI depends on the number of Dd and the number of Mm. The former is not directly quantifiable since the cells must remain adherent for the infection; instead, it is estimated to be in the range of 3–4*10^7^ cells per 80–90% confluent Petri dish from previous experiments, combined with visual inspection of the confluency. The standard and previously published protocols (Mottet et al., 2021) typically use an MOI of 10, which has consistently resulted in robust and reproducible infection, affecting approximately 50% of the amoebae with an average of 1 to 5 bacteria per cell.

#### Note for Mm ΔRD1mutant

Some Mm mutants are attenuated during infection and may require an adjustment of the MOI; for example, the ΔRD1 mutant, for which the MOI is doubled to compensate for its reduced phagocytosis. Due to the higher MOI used for such mutants, additional washes are necessary to eliminate extracellular bacteria.

### Monitoring the course of infection

#### Intracellular growth of *Mycobacterium marinum* during infection

**Figure 1:**
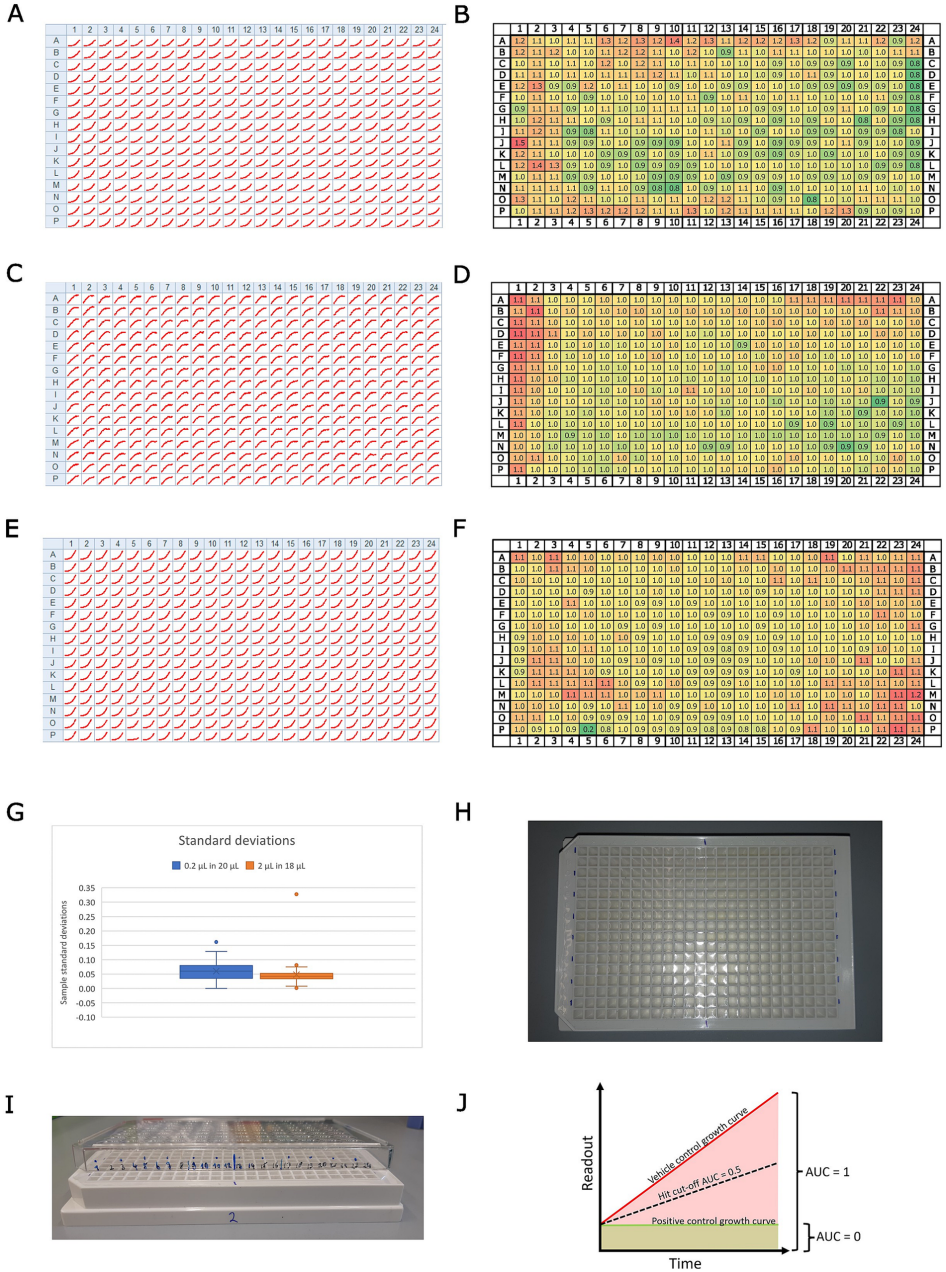
Overview of the assay design. Panels **(A,C,E)** show growth curves for every position on a 384-well plate for **(A)**, Mm in infection; **(C)**, Dd in infection; and **(E)**, Mm in broth. The homogeneity of these results across the full plate was assessed by dividing the maximum raw value of each well by the median of all maxima. The resulting rounded fold change is depicted in panels **(B)**, Mm in infection; **(D)**, Dd in infection; and **(F)**, Mm in broth. The color code indicates low values with green and high values with red tones. Panel **(G)** shows a box plot with the standard deviations of a set of about 50 compounds, which were tested at the same concentration on Mm in broth, once by addition in a 1:100 dilution (0.2 μl in 20 μl, blue) and once by addition in a 1:10 dilution (2 μl in 18 μl, orange). Panel **(H)** shows a photo illustrating the labeling of the plate for easier pipetting. Panel **(I)** shows a photo of the same plate with a labeled lid on top, illustrating a method to keep track of pipetted wells. Panel **(J)** illustrates the calculation of the normalized residual growth (NRG). The area under the curve of the vehicle control is scaled to 1, while the area under the curve of the positive control is scaled to 0.

- To prepare cells for a growth assay in a 96-well plate (luminescence: Thermo Scientific 136101) or 384-well plates (luminescence: Interchim FP-BA8240) using Mm LuxCDABE (Andreu et al., 2010) or Mm pMSP12: GFP (Addgene plasmid # 30167) (Davis et al., 2002), measure cell density at the Countess and prepare the final cell suspension via a 1:10 intermediate dilution.
- In 96-well plates, 2.5*10^5^ cells/ml (5*10^4^ cells per well in 200 μl) are seeded.
- In 384 opaque-bottomed well plates (Interchim FP-BA8240), 5*10^4^ cells/ml (10^3^ cells per well in 20 μl) are seeded.
- Prepare a cell suspension (approximately 20 ml per 96-well plate, approximately 10 ml per 384-well plate) at the correct density in a conical tube. Cells can be plated using a multipipette (Sartorius Picus, 12 channel, 5–120 μl) or an automatic dispenser.
- For 96-well plates, it is recommended to fill the inter-well space with 150 μl of double-distilled, sterile water, thereby minimizing the “edge effect” of extreme growth at the edge of the well plate (see Figures 1A–F).
- It is recommended to briefly spin down 384-well plates to ensure the liquid is at the bottom of the well, both after plating cells and after adding test compounds.
- Add test compounds in a 1:100 dilution: 0.2 μl in 20 μl, ideally using an electronic multi-pipette (see “Note on compound addition,” Figure 1G).
- Seal plates with a gas-impermeable (ROTILABO, H769.1; 384 WPs) or gas-permeable seal (4titude, 4ti-0516/96; 96 WPs). Since the airtight seals come non-sterile, it is recommended to sterilize them using the UV program of one of the laminar flow benches.
- To limit the edge effect of stacked plates, it is recommended to place an empty, unsealed dummy plate at the first and last positions of the stack.
- Luminescence and fluorescence are read from the top, with gain values signaling saturation (at a gain of circa 100 for both readouts with a BioTek Synergy H1 reader) and an integration time of 1 s (higher integration time does not impact RLU or RFU but decreases the noise of the measurement). If time resolution is not a concern, 10s of integration time is recommended.

##### Note on using a stacker and controlling the temperature

When using the BioStack, the assay plates spend most of the 72 h of the assay in the stacker. Consequently, the ambient temperature should be controlled to ensure the growth of bacteria and amoebae. Both organisms have different temperature optima, and the optimal compromise is at 25°C. However, the BioTek Synergy H1 plate reader is placed in the same environment as the BioStack and can heat but not cool the space around the plate carrier. Consequently, the inside of the plate reader will reach temperatures of around 30°C. To avoid such a temperature shock to the amoebae, we set the ambient temperature to 24°C, allowing for growth while the samples are in the stacker and minimizing heat shock during measurement.

##### Note on compound addition

Different strategies were tested for optimal addition of the compounds to 384-well plates: 1. A dispenser (Agilent Bravo liquid handler) in a 1:10 dilution, 2. an electronic multi pipette (Sartorius Picus, 12 channel, 0.2–10 μl) in a 1:10 dilution, 3. a 1:100 dilution, and 4. a single-channel pipette (Gilson, P2) used with a 1:100 dilution. Options 2 and 3, i.e., using an electronic multi-pipette with a 1:100 or 1:10 dilution (i.e., adding 0.2 μl in 20 μl or 2 μl in 20 μl, respectively), are preferable due to good control over the added volume. The box plot in Figure 1G illustrates the distribution of standard deviations for a set of approximately 50 compounds tested at the same final concentration against Mm in broth, using the two different dilution formats: a 1:100 dilution (0.2 μl in 20 μl, shown in blue) and a 1:10 dilution (2 μl in 18 μl; shown in orange). Notably, the obtained results showed a negligible difference in measurement scatter between using a 1:10 or a 1:100 dilution (Figure 1G). Consequently, we preferred a 1:100 pipetting regime, as it provided us with more flexibility in handling stocks of test conditions. Additionally, we recommend a comprehensive well plate design. For example, we used sector four (i.e., every bottom right well of a quadruple well) exclusively for controls. Generally, vehicle controls were homogeneously distributed across the plate, with the positive control (10 μM rifabutin) located in the top and bottom rows. At least three biological replicates were acquired (i.e., different Petri dishes of infected amoebae) with 3, 2, or 1 technical replicate each, depending on the availability of the extracts or compounds.

#### Growth of *Mycobacterium marinum* LuxCDABE in broth

- Prepare the Mm culture as described for the infection.
- On the experiment day, directly take 1 ml of the culture supernatant (after centrifuging for 1 min at 300 rpm, as for the infection) and transfer it to an Eppendorf tube.
- Homogenize the culture using a syringe as described in the infection protocol.
- Measure the optical density (OD) of the syringe-cultured supernatant as described previously.
- Calculate the required volume of culture supernatant to prepare a suspension of 3.75*10^5^ bacteria/ml (7,500 bacteria per well in 20 μl).
- Prepare this suspension by diluting the syringed culture supernatant 1:10 (900 μl 7H9 + 100 μl culture supernatant) to reduce the margin of error associated with pipetting small volumes.
- Fill 384-well plates with an electronic multi-pipette and centrifuge briefly before adding test extracts, compounds, or mixtures.
- For the use of the stacker or plate reader, follow the procedure described above for the infection assay.

#### Note on using a stacker and controlling the temperature

As described above, the fact that the plate reader cannot cool its internal temperature while being placed in a heated environment can lead to a heat shock for the samples. In the case of the “in broth” assay, we set the ambient temperature to 27°C, resulting in an approximate temperature of 30°C inside the plate reader. These temperatures are indeed below the optimal growth temperature of Mm but were observed to dampen an edge effect by sacrificing overall growth.

## Results

### Controlling and measuring the edge effect of plate reader assays

In this high-throughput screening (HTS) experiment, we implemented specific measures to minimize and quantify the edge effect, a common issue in microplate assays where wells at the edges experience different environmental conditions (e.g., evaporation and temperature fluctuations) compared to inner wells. To mitigate this effect, we lowered the temperature of the incubation box and reduced the initial inoculum size, thereby improving consistency across wells. To assess the uniformity of results across the 384-well plate, we analyzed the growth curves for each well position (see Figure 1). Panels A, C, and E in Figure 1 present growth curves for different conditions: (A) Mm during infection, (C) Dd during infection, and (E) Mm in broth. To quantify plate-wide homogeneity, we normalized the maximum raw value of each well by dividing it by the median of all maxima. This method helps account for variations due to edge effects or other inconsistencies. The resulting rounded fold change values are illustrated in Figure 1, panels B (Mm during infection), D (Dd during infection), and F (Mm in broth), allowing for a direct visual comparison of data distribution across the plate. In addition, the scalability of the HTS was evaluated by testing.

### Data analysis

Data obtained from BioTek Synergy H1 plate readers from infection or broth assays are processed analogously using custom scripts written in R (Supplementary File S1) and are publicly available (Git Hub, 2024). First, every well is annotated with the name of the test condition, the respective test concentration, the biological replicate as a number, and the corresponding vehicle control. Vehicle control wells are flagged with the prefix “VC,” and positive controls with the prefix “PC.” Then, annotated raw data are submitted to R to generate summary data. Briefly, the script iterates over well plates saved as separate xlsx files, extracts RLU and RFU values for each well, and calculates the area under the curve (AUC) for each well. Subsequently, it calculates the median, median absolute deviation (MAD), the mean, and standard deviation of the AUC for all vehicle controls. Additionally, we compute the median of the first measurement across all conditions of a well plate and extrapolate it over the entire time course of the experiment to calculate the AUC of this baseline. These two values are used to linearly scale test conditions between 0 (the baseline AUC) and 1 (the vehicle control AUC), yielding one summary value per well (see Figure 1J). Additionally, the robust Z’-factor (Atmaramani et al., 2020) is calculated for each vehicle control on each plate by using the respective vehicle control and the positive control as a reference. A PDF report containing plots, such as the temperature over time or heatmaps sorted by test condition, is helpful for troubleshooting and visualization. In the second step, biological replicates are integrated by looping over the respective summary data, notably the scaled AUC, and calculating the median overall technical replicates in all biological replicates for each condition. This workflow of normalized AUC is applied to both readouts, RFU and RLU, in infection and to RLU in broth to obtain a comparable and quantitative measure, which we call “normalized residual growth” (NRG). This allows for the establishment of a hard cut-off for fast-hit classification on every readout. Based on our experience and the observed scatter of test conditions, we deemed an NRG of 0.5 a robust cut-off (see Figure 1J). The NRG can take negative values in the case of a growth curve with a negative slope, i.e., decreasing below the starting value, indicating cell or bacterial killing, which leads to an AUC below the extrapolated baseline. However, the assay is intended as a growth inhibition assay and not a killing assay, with the highest resolution between the two references. A PDF report, including plots that compare technical replicates per biological replicate in a heatmap or correlate the scaled AUC with the AUC, is helpful for troubleshooting and visualization. In the case of dose–response curves (DRCs), plots with the log_10_ of the test concentration on the x-axis and the scaled area under the curve (AUC) on the y-axis provide a quick overview of the tested conditions. The half-inhibitory concentration (IC_50_) was calculated using GraphPad Prism (Version 8.0.1) with NRG, employing a robust 4PL regression, constraining the top value to 1 and, if necessary, the bottom value to −2 (Sebaugh, 2011). A minimum inhibitory concentration (MIC) was determined as the lowest experimental concentration at which the averaged normalized residual growth, plus or minus one standard deviation, overlapped with 0 (Magréault et al., 2022).

### Benchmarking of the Dd-Mm high-throughput platform

**Figure 2:**
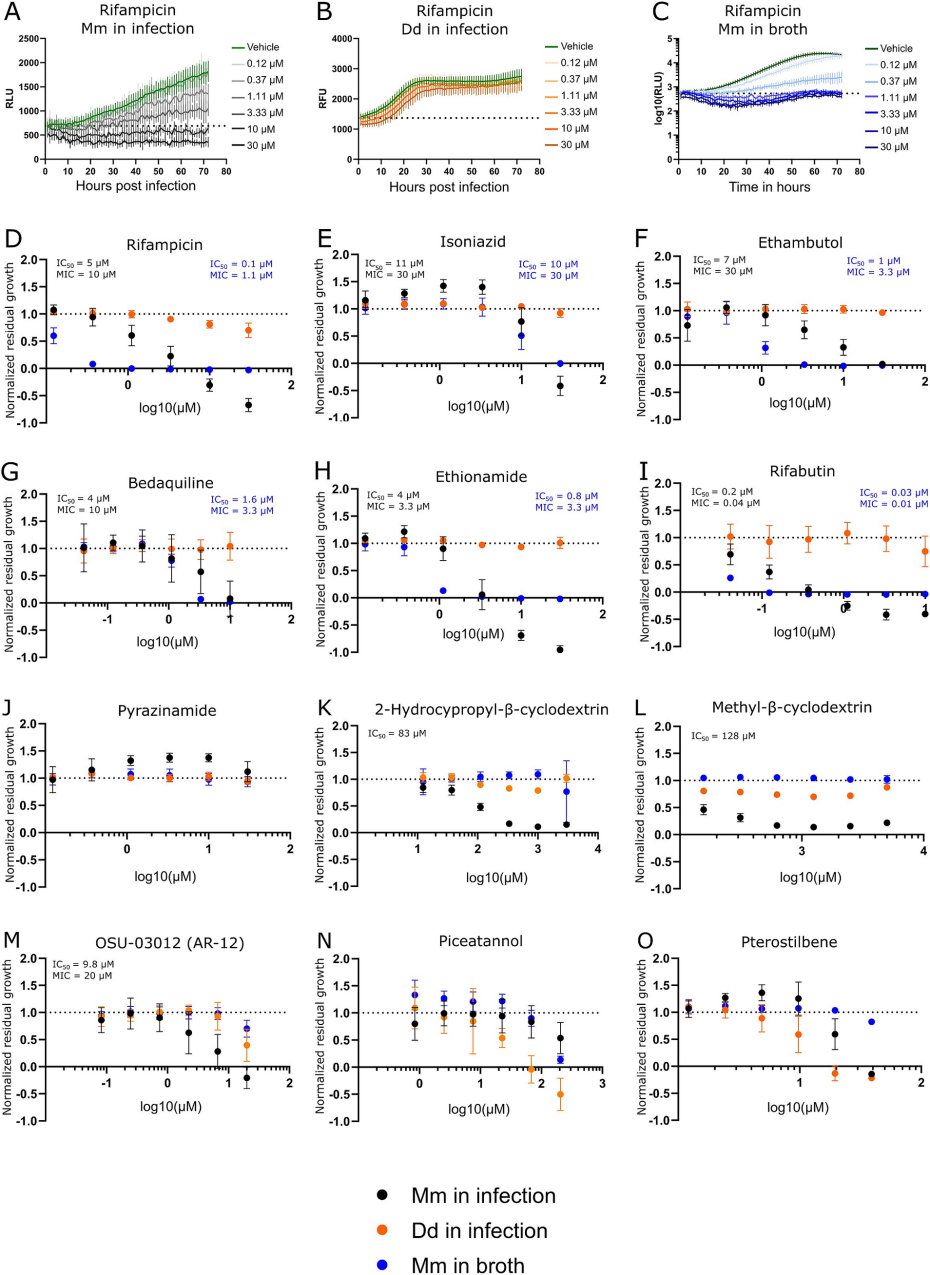
Dose–response curves of benchmarked antibiotics, anti-infectives, and Dd inhibitors. A selection of antibiotics used for the treatment of a TB infection was benchmarked in the Dd-Mm high-throughput system, in infection, and in broth. In panels **(A,B,C)**, 72-h growth curves of Mm in infection, Dd in infection, and Mm in broth, respectively, at different concentrations of rifampicin are shown. The dashed line represents the median over the first measurements of all wells of the respective experiment. On the y-axis are random luminescence units (RLU) or random fluorescence units (RFU). The former were log10 transformed in panel **(C)**. The corresponding dose–response curve of rifampicin is shown in panel **(D)**. The dose–response curves depicted in panels **(E)** (isoniazid), **(F)** (ethambutol), **(G)** (bedaquiline), **(H)** (ethionamide), **(I)** (rifabutin) and **(J)** (pyrazinamide) was created from growth curves in an analogous manner. Anti-infective compounds were benchmarked in panels **(K)** (2-hydroxypropyl-β-cyclodextrin), **(L)** (methyl-β-cyclodextrin), and **(M)** (OSU-03012 or AR-12). Panels **(N,O)** show the stilbenes piceatannol and pterostilbene, respectively, both Dd growth inhibitors. Black points show Mm in infection. Orange points Dd in infection, and blue points Mm in broth. Log10 of the test concentration in μM is shown on the x-axis. A dashed line at y = 1 represents the normalized residual growth of the vehicle control. Depicted are means of at least three biological replicates with at least two technical replicates each and the respective standard deviations. Black and blue text inlays show the MIC and IC50 of the anti-infective or the antibacterial assay, respectively.

The high-throughput *Dictyostelium discoideum*-*Mycobacterium marinum* (Dd-Mm) infection model was benchmarked with selected antibiotics commonly used in tuberculosis (TB) treatment and anti-infectives by generating dose–response curves (DRCs) and calculating IC_50_s and MICs where applicable (Figure 2). The benchmarking involved two complementary assays. First, an anti-infective assay measured the intracellular growth of a bioluminescent Mm within Dd cells expressing mCherry. This setup also enabled simultaneous monitoring of the host amoebae’s growth. Second, an antibiotic assay was used to assess Mm growth in broth. In both assays, luminescence served as an indicator of bacterial biomass and metabolic activity. Bacteria or infected amoebae were plated in 384-well plates, treated with decreasing concentrations of each antibiotic, and monitored over 72 h using a plate reader.

We observed susceptibility to first-line antibiotics used against TB, including rifampicin, ethambutol, and isoniazid, but not to pyrazinamide (see Figures 2A–F,J). Indeed, Mm is reported to be naturally resistant to pyrazinamide (Aubry et al., 2000; Wagner and Young, 2004). In other susceptibility tests, it was also reported that Mm was susceptible to ethambutol and isoniazid only above certain breaking points (Aubry et al., 2000). Susceptibility to both antibiotics was observed, however, notably for isoniazid at a relatively high concentration in infection and broth (MIC of 30 μM). Furthermore, susceptibility to bedaquiline, ethionamide, and rifabutin, an analog of rifampin, was also detected (see Figures 2G–I). The Dd-Mm system was also benchmarked with compounds that have higher activity in infection compared to those in broth, which we classify as “strict anti-infective.” For example, methyl-β-cyclodextrin and 2-hydroxypropyl-β-cyclodextrin reduced Mm growth in infected mice but not in broth (see Figures 2K,L). In parallel, OSU-03012 (AR-12), a drug used as an autophagy inducer, was tested and appears to act as a strict anti-infective, but only within a specific and narrow dose range (Figure 2M) (Chiu et al., 2009; Gao et al., 2008). We perturbed Dd growth with the two stilbenes, piceatannol and pterostilbene. We demonstrated that within a dose range, Dd growth can be severely limited while still allowing for almost unperturbed Mm growth (see Figures 2N,O), indicating that Dd growth and intracellular Mm growth are partially independent. The average robust Z’-factor of the respective benchmarking experiments was 0.73 for Mm in infection and 0.61 for Mm in broth. Overall, we demonstrated the robustness of the Dd-Mm high through-put platform and its capability to differentiate between antibiotics and anti-infective compounds.

## Discussion

The data processing pipeline developed for the Dd-Mm infection and broth assays provides a robust and reproducible framework for analyzing high-throughput screening experiments. This workflow, implemented using custom R scripts, effectively automates data annotation, normalization, and summary generation, ensuring consistency and scalability in handling large datasets. Key steps in the pipeline include annotating test conditions, concentrations, and controls, followed by calculating the area under the curve (AUC) for luminescence (RLU) and fluorescence (RFU) measurements. By scaling the AUC between the baseline and vehicle control values, the pipeline generates a normalized residual growth (NRG) metric, providing a standardized quantitative readout across plates. This normalization facilitates direct comparison between infection and broth assays, as well as across biological replicates, thereby enhancing the reliability of the results. The inclusion of robust statistical measures, such as the median absolute deviation (MAD) and the Z’-factor, ensures the quality of the data. The Z’-factor calculation, using vehicle and positive controls as references, validates assay robustness and helps identify variability, a critical feature for high-throughput settings. Overall, this workflow represents a powerful and adaptable tool for evaluating anti-infective compounds in high-throughput assays. Its ability to integrate technical and biological replicates, normalize data across experimental conditions, and provide clear visualization supports confident decision-making in drug discovery. Future refinements could include expanding its application to killing assays or integrating machine learning algorithms for automated hit classification and outlier detection. The systematic and quantitative nature of this pipeline underscores its utility in high-throughput infection model platforms.

The susceptibility profile observed in the Dd-Mm infection model aligns well with known characteristics of Mm and its relationship to TB treatment. Susceptibility to first-line TB antibiotics rifampicin, ethambutol, and isoniazid was confirmed, with the exception of pyrazinamide, confirming previous findings that Mm is naturally resistant to this drug The platform’s versatility was further highlighted by its ability to detect susceptibility to additional TB-relevant antibiotics, including bedaquiline, ethionamide, and rifabutin, a rifampicin analog. Notably, bedaquiline demonstrated not only its antibiotic activity but also potential host-beneficial effects, as previously reported (Giraud-Gatineau et al., 2020). These dual effects make it an attractive candidate for further investigation into host-pathogen dynamics. The benchmarking of compounds with higher activity in infection compared to broth—classified as “strict anti-infectives”—demonstrates the Dd-Mm system’s capability to distinguish between general antibiotics and compounds with infection-specific activity. For instance, methyl-β-cyclodextrin and 2-hydroxypropyl-β-cyclodextrin reduced Mm growth exclusively in the infection model, suggesting that these compounds may target host–pathogen interactions or intracellular bacterial survival mechanisms. Cyclodextrins have been reported as intrinsically bactericidal, which we did not observe at the tested doses. However, they are also speculated to manipulate the host-pathogen interface by depleting host membranes of sterol (Hammoud et al., 2019). Similarly, OSU-03012 (AR-12), an autophagy inducer, exhibited strict anti-infective activity within a narrow dose range, underscoring the platform’s sensitivity in detecting dose-dependent effects. Overall, the Dd-Mm system represents a powerful tool for investigating not only host–pathogen interactions but also for distinguishing between antibiotics and anti-infective compounds, further underscoring its utility in drug discovery and potentially identifying novel therapeutic strategies targeting intracellular pathogens.

While Mm shares many genetic and pathogenic features with Mtb, important differences must be considered when using Mm as a model organism. One key distinction lies in their drug susceptibility profiles. Both species are sensitive to core antimycobacterial agents such as rifampicin and clarithromycin; however, Mm shows reduced susceptibility to isoniazid and variable responses to ethambutol, likely due to differences in cell wall composition and metabolic pathways. Despite these differences, numerous reviews have highlighted Mm as a relevant and valuable model for TB drug discovery (Antunes et al., 2024). We benchmarked our high-throughput Dd–Mm infection platform with seven standard anti-TB drugs and found that all MICs fell below the NCCLS breakpoints for Mm, except for ethambutol in the infection model (30 μM or 6.1 μg/ml). These results support the platform’s reliability for screening anti-mycobacterial drugs. Interestingly, both ethambutol and rifampicin showed reduced efficacy during infection compared to broth culture—an effect similarly observed in RAW264.7 and THP-1 macrophages (Christophe et al., 2010; Nitschke et al., 2024). In parallel, Sorrentino et al. evaluated 144 compounds from the GSK TB set in *Mtb*-infected THP-1 cells using a luciferase reporter, confirming intracellular activity for 90.3% of the compounds (Sorrentino et al., 2016). We tested the same compound set in our lab, and notably, 8 of the top 10 hits identified by Sorrentino et al. also demonstrated high potency in our assays (Trofimov et al., 2018), underscoring the complementarity and robustness of the two platforms.

A recent high-throughput assay screened 28,000 compounds against Mm to identify both antibacterial and antivirulence agents. This screen yielded 11 hits, of which seven were validated as virulence inhibitors and one as a bactericidal compound in Mtb, highlighting the potential of this model-based approach for TB drug discovery (Tükenmez et al., 2019). In terms of intracellular behavior, both species are facultative intracellular pathogens capable of surviving and replicating within macrophages. However, Mtb is highly adapted to long-term persistence and latency in human macrophages, whereas Mm typically induces more rapid granuloma formation and is more inflammatory in animal models such as zebrafish. Despite these differences, the shared mechanisms of intracellular survival and immune evasion still make Mm a useful proxy for studying key aspects of mycobacterial pathogenesis and drug response (Cardenal-Munoz et al., 2018; Guallar-Garrido and Soldati, 2024).

On the host side, Dd shares with macrophages a core set of antimicrobial responses, including a functional phagosome maturation pathway, production of reactive oxygen species (ROS) via NADPH oxidase-like systems, and metal-based defenses such as zinc poisoning through ZnT transporters and iron depletion through NRAMP family transporters. The amoeba also mounts robust autophagy and xenophagy responses, which are essential for controlling intracellular pathogens (Guallar-Garrido and Soldati, 2024). Although Dd offers a valuable model for studying host–pathogen interactions and innate immune responses, it presents several limitations when used to test anti-infective compounds compared to mammalian systems. Notably, as a single-cell amoeba and like any macrophage or other tissue-cultured cell, it lacks an adaptive immune system, making it unsuitable for evaluating compounds that target or rely on T- or B-cell-mediated responses. Although complex organisms can recapitulate the infection dynamics of Mtb and macrophages, the unicellular nature and absence of complex tissues or organ systems prevent the modeling of tissue-specific infection dynamics and systemic drug effects. Key mammalian immune signaling pathways, such as NF-κB and interferon responses, are absent or highly divergent, reducing the model’s predictive power for host-directed therapies. Therefore, while useful for initial screening and mechanistic studies, findings in Dd typically require validation in animal models to assess clinical potential.

## Data Availability

The original contributions presented in the study are included in the article/Supplementary material. Further inquiries can be directed to the corresponding author.

## References

[ref1] Altaf M. Miller C. H. Bellows D. S. O’Toole R. (2010). Evaluation of the Mycobacterium smegmatis and BCG models for the discovery of *Mycobacterium tuberculosis* inhibitors. Tuberculosis 90, 333–337. doi: 10.1016/j.tube.2010.09.002, PMID: 20933470

[ref2] Andreu N. Zelmer A. Fletcher T. Elkington P. T. Ward T. H. Ripoll J. . (2010). Optimisation of bioluminescent reporters for use with mycobacteria. PLoS One 5:e10777. doi: 10.1371/journal.pone.0010777, PMID: 20520722 PMC2875389

[ref3] Andries K. Verhasselt P. Guillemont J. Göhlmann H. W. H. Neefs J.-M. Winkler H. . (2005). A Diarylquinoline drug active on the ATP synthase of *Mycobacterium tuberculosis*. Science 307, 223–227. doi: 10.1126/science.1106753, PMID: 15591164

[ref4] Antunes S. S. Forn-Cuní G. Romeiro N. C. Spaink H. P. Verbeek F. J. Muzitano M. F. (2024). Embryonic and larval zebrafish models for the discovery of new bioactive compounds against tuberculosis. Drug Discov. Today 29:104163. doi: 10.1016/j.drudis.2024.104163, PMID: 39245344

[ref5] Arafah S. Kicka S. Trofimov V. Hagedorn M. Andreu N. Wiles S. . (2013). “Setting up and monitoring an infection of Dictyostelium discoideum with mycobacteria” in Dictyostelium discoideum Protocols. Eds. Ludwig Eichinger, Francisco Rivero (Totowa, NJ: Humana Press), 403–417. 10.1007/978-1-62703-302-2_22 23494320

[ref6] Atmaramani R. Pancrazio J. J. Black B. J. (2020). Adaptation of robust Z’ factor for assay quality assessment in microelectrode array based screening using adult dorsal root ganglion neurons. J. Neurosci. Methods 339:108699. doi: 10.1016/j.jneumeth.2020.108699, PMID: 32224158 PMC11808661

[ref7] Aubry A. Jarlier V. Escolano S. Truffot-Pernot C. Cambau E. (2000). Antibiotic susceptibility pattern of *Mycobacterium marinum*. Antimicrob. Agents Chemother. 44, 3133–3136. doi: 10.1128/AAC.44.11.3133-3136.2000, PMID: 11036036 PMC101616

[ref8] Batt S. M. Minnikin D. E. Besra G. S. (2020). The thick waxy coat of mycobacteria, a protective layer against antibiotics and the host’s immune system. Biochem. J. 477, 1983–2006. doi: 10.1042/BCJ20200194, PMID: 32470138 PMC7261415

[ref9] Bloomfield G. Tanaka Y. Skelton J. Ivens A. Kay R. R. (2008). Widespread duplications in the genomes of laboratory stocks of Dictyostelium discoideum. Genome Biol. 9:R75. doi: 10.1186/gb-2008-9-4-r75, PMID: 18430225 PMC2643946

[ref10] Calmette A Guérin C Boquet A Nègre L. (1927). La vaccination préventive contre la tuberculose par le" BCG, ". Masson et cie.

[ref11] Cardenal-Munoz E. Barisch C. Lefrancois L. H. Lopez-Jimenez A. T. Soldati T. (2018). When Dicty met Myco, a (not so) romantic story about one Amoeba and its intracellular pathogen. Front. Cell. Infect. Microbiol. 7:529. doi: 10.3389/fcimb.2017.00529, PMID: 29376033 PMC5767268

[ref12] Chiu H.-C. Soni S. Kulp S. K. Curry H. Wang D. Gunn J. S. . (2009). Eradication of intracellular *Francisella tularensis* in THP-1 human macrophages with a novel autophagy inducing agent. J. Biomed. Sci. 16:110. doi: 10.1186/1423-0127-16-110, PMID: 20003180 PMC2801672

[ref13] Christophe T. Ewann F. Jeon H. K. Cechetto J. Brodin P. (2010). High-content imaging of *Mycobacterium tuberculosis* -infected macrophages: an in vitro model for tuberculosis drug discovery. Future Med. Chem. 2, 1283–1293. doi: 10.4155/fmc.10.223, PMID: 21426019

[ref14] Davis J. M. Clay H. Lewis J. L. Ghori N. Herbomel P. Ramakrishnan L. (2002). Real-time visualization of Mycobacterium-macrophage interactions leading to initiation of granuloma formation in zebrafish embryos. Immunity 17, 693–702. doi: 10.1016/S1074-7613(02)00475-2, PMID: 12479816

[ref15] Dunn J. D. Bosmani C. Barisch C. Raykov L. Lefrançois L. H. Cardenal-Muñoz E. . (2018). Eat prey, live: Dictyostelium discoideum as a model for cell-autonomous defenses. Front. Immunol. 8:1906. doi: 10.3389/fimmu.2017.01906, PMID: 29354124 PMC5758549

[ref16] Eichinger L. Pachebat J. A. Glöckner G. Rajandream M.-A. Sucgang R. Berriman M. . (2005). The genome of the social amoeba Dictyostelium discoideum. Nature 435, 43–57. doi: 10.1038/nature03481, PMID: 15875012 PMC1352341

[ref17] Fey P. Kowal A. S. Gaudet P. Pilcher K. E. Chisholm R. L. (2007). Protocols for growth and development of Dictyostelium discoideum. Nat. Protoc. 2, 1307–1316. doi: 10.1038/nprot.2007.178, PMID: 17545967

[ref18] Gao M. Yeh P. Y. Lu Y.-S. Hsu C.-H. Chen K.-F. Lee W.-C. . (2008). OSU-03012, a novel celecoxib derivative, induces reactive oxygen species–related autophagy in hepatocellular carcinoma. Cancer Res. 68, 9348–9357. doi: 10.1158/0008-5472.CAN-08-1642, PMID: 19010909

[ref19] Giraud-Gatineau A. Coya J. M. Maure A. Biton A. Thomson M. Bernard E. M. . (2020). The antibiotic bedaquiline activates host macrophage innate immune resistance to bacterial infection. eLife 9:e55692. doi: 10.7554/eLife.55692, PMID: 32369020 PMC7200153

[ref20] Git Hub. (2024). Jahn Nitschke/Dd-mm-growth-assay-analysis: Illustratory R scripts used for data analyses of high content plate reader screens of the Dd-mm system. Available online at: https://github.com/JahnNitschke/Dd-Mm-growth-assay-analysis (Accessed February 17, 2024).

[ref21] Guallar-Garrido S. Soldati T. (2024). Exploring the relevance of the *Dictyostelium discoideum*-*Mycobacterium marinum* infection model for tuberculosis research. Dis. Model. Mech. 17:698. doi: 10.1242/dmm.050698, PMID: 39037280 PMC11552500

[ref22] Hammoud Z. Khreich N. Auezova L. Fourmentin S. Elaissari A. Greige-Gerges H. (2019). Cyclodextrin-membrane interaction in drug delivery and membrane structure maintenance. Int. J. Pharm. 564, 59–76. doi: 10.1016/j.ijpharm.2019.03.063, PMID: 30959238

[ref23] Hanna N Burdet F Melotti A Bosmani C Kicka S Hilbi H . (2019). Time-resolved RNA-seq profiling of the infection of Dictyostelium discoideum by *Mycobacterium marinum* reveals an integrated host response to damage and stress. Syst. Biol. [Preprint]. doi: 10.1101/590810

[ref24] Hanna N. Kicka S. Chiriano G. Harrison C. Sakouhi H. O. Trofimov V. . (2020). Identification of anti-Mycobacterium and anti-Legionella compounds with potential distinctive structural scaffolds from an HD-PBL using phenotypic screens in amoebae host models. Front. Microbiol. 11:266. doi: 10.3389/fmicb.2020.00266, PMID: 32153546 PMC7047896

[ref25] Kanabalan R. D. Lee L. J. Lee T. Y. Chong P. P. Hassan L. Ismail R. . (2021). Human tuberculosis and *Mycobacterium tuberculosis* complex: a review on genetic diversity, pathogenesis and omics approaches in host biomarkers discovery. Microbiol. Res. 246:126674. doi: 10.1016/j.micres.2020.126674, PMID: 33549960

[ref26] Kirchhoffer O. A. Nitschke J. Allard P.-M. Marcourt L. David B. Grondin A. . (2023). Targeted isolation of natural analogs of anti-mycobacterial hit compounds based on the metabolite profiling of a large collection of plant extracts. Front. Nat. Prod. 2:1279761. doi: 10.3389/fntpr.2023.1279761

[ref27] Kirchhoffer OA Quiros-Guerrero L Nitschke J Nothias L-F Burdet F Marcourt L . (2024). Prioritization of Novel Anti-infective Stilbene derivatives by Combining Metabolomic Data Organization and a Stringent 3R-infection Model in a Knowledge Graph. 10.1039/d4ra08421g PMC12015462 40271414

[ref28] Kuspa A. (2006). “Restriction enzyme-mediated integration (REMI) mutagenesis” in *Dictyostelium discoideum* Protocols. Eds. Ludwig Eichinger, Francisco Rivero (New Jersey: Humana Press), 201–210. 10.1385/1-59745-144-4:201 16957292

[ref29] Lefrançois L. H. Nitschke J. Wu H. Panis G. Prados J. Butler R. E. . (2024). Temporal genome-wide fitness analysis of *Mycobacterium marinum* during infection reveals the genetic requirement for virulence and survival in amoebae and microglial cells. mSystems 9, e01326–e01323. doi: 10.1128/msystems.01326-23 38270456 PMC10878075

[ref30] López-Jiménez A. T. Cardenal-Muñoz E. Leuba F. Gerstenmaier L. Barisch C. Hagedorn M. . (2018). The ESCRT and autophagy machineries cooperate to repair ESX-1-dependent damage at the Mycobacterium-containing vacuole but have opposite impact on containing the infection. PLoS Pathog. 14:e1007501. doi: 10.1371/journal.ppat.1007501, PMID: 30596802 PMC6329560

[ref31] Magréault S. Jauréguy F. Carbonnelle E. Zahar J.-R. (2022). When and how to use MIC in clinical practice? Antibiotics 11:1748. doi: 10.3390/antibiotics11121748, PMID: 36551405 PMC9774413

[ref32] Mottet M. Bosmani C. Hanna N. Nitschke J. Lefrançois L. H. Soldati T. (2021). Novel single-cell and high-throughput microscopy techniques to monitor Dictyostelium discoideum-*Mycobacterium marinum* infection dynamics. Methods Mol. Biol. 2314, 183–203. doi: 10.1007/978-1-0716-1460-0_7, PMID: 34235653

[ref33] Nitschke J. Huber R. Vossio S. Moreau D. Marcourt L. Gindro K. . (2024). Discovery of anti-infective compounds against *Mycobacterium marinum* after biotransformation of simple natural stilbenes by a fungal secretome. Front. Microbiol. 15:1439814. doi: 10.3389/fmicb.2024.1439814, PMID: 39355425 PMC11443511

[ref34] Pethe K. Bifani P. Jang J. Kang S. Park S. Ahn S. . (2013). Discovery of Q203, a potent clinical candidate for the treatment of tuberculosis. Nat. Med. 19, 1157–1160. doi: 10.1038/nm.3262, PMID: 23913123

[ref35] Ramakrishnan L. (2004). Using Mycobacterium marinum and its hosts to study tuberculosis. Curr. Sci. 86, 82–92.

[ref36] Raykov L. Mottet M. Nitschke J. Soldati T. (2023). A TRAF-like E3 ubiquitin ligase Traf E coordinates ESCRT and autophagy in endolysosomal damage response and cell-autonomous immunity to *Mycobacterium marinum*. eLife 12:e85727. doi: 10.7554/eLife.85727, PMID: 37070811 PMC10181826

[ref37] Schaaf K. Hayley V. Speer A. Wolschendorf F. Niederweis M. Kutsch O. . (2016). A macrophage infection model to predict drug efficacy against *Mycobacterium Tuberculosis*. Assay Drug Dev. Technol. 14, 345–354. doi: 10.1089/adt.2016.717, PMID: 27327048 PMC4991579

[ref38] Sebaugh J. L. (2011). Guidelines for accurate EC50/IC50 estimation. Pharm. Stat. 10, 128–134. doi: 10.1002/pst.426, PMID: 22328315

[ref39] Solomon J. M. Isberg R. R. (2000). Growth of *Legionella pneumophila* in Dictyostelium discoideum: a novel system for genetic analysis of host–pathogen interactions. Trends Microbiol. 8, 478–480. doi: 10.1016/S0966-842X(00)01852-7, PMID: 11044684

[ref40] Sorrentino F. Gonzalez del Rio R. Zheng X. Presa Matilla J. Torres Gomez P. Martinez Hoyos M. . (2016). Development of an intracellular screen for new compounds able to inhibit *Mycobacterium tuberculosis* growth in human macrophages. Antimicrob. Agents Chemother. 60, 640–645. doi: 10.1128/AAC.01920-15, PMID: 26503663 PMC4704166

[ref41] Stinear T. P. Seemann T. Harrison P. F. Jenkin G. A. Davies J. K. Johnson P. D. R. . (2008). Insights from the complete genome sequence of *Mycobacterium marinum* on the evolution of *Mycobacterium tuberculosis*. Genome Res. 18, 729–741. doi: 10.1101/gr.075069.107, PMID: 18403782 PMC2336800

[ref42] Taneja N. K. Tyagi J. S. (2007). Resazurin reduction assays for screening of anti-tubercular compounds against dormant and actively growing *Mycobacterium tuberculosis*, *Mycobacterium bovis* BCG and *Mycobacterium smegmatis*. J. Antimicrob. Chemother. 60, 288–293. doi: 10.1093/jac/dkm207, PMID: 17586560

[ref43] Tobin D. M. (2015). Host-directed therapies for tuberculosis. Cold Spring Harb. Perspect. Med. 5:a021196. doi: 10.1101/cshperspect.a021196, PMID: 25986592 PMC4588138

[ref44] Tobin D. M. Ramakrishnan L. (2008). Comparative pathogenesis of Mycobacterium marinum and *Mycobacterium tuberculosis*. Cell. Microbiol. 10, 1027–1039. doi: 10.1111/j.1462-5822.2008.01133.x, PMID: 18298637

[ref45] Trofimov V. Kicka S. Mucaria S. Hanna N. Ramon-Olayo F. Del Peral L. V.-G. . (2018). Antimycobacterial drug discovery using mycobacteria-infected amoebae identifies anti-infectives and new molecular targets. Sci. Rep. 8:3939. doi: 10.1038/s41598-018-22228-6, PMID: 29500372 PMC5834492

[ref46] Tükenmez H. Edström I. Ummanni R. Fick S. B. Sundin C. Elofsson M. . (2019). *Mycobacterium tuberculosis* virulence inhibitors discovered by *Mycobacterium marinum* high-throughput screening. Sci. Rep. 9:26. doi: 10.1038/s41598-018-37176-4, PMID: 30631100 PMC6328581

[ref47] Udinia S. Suar M. Kumar D. (2023). Host-directed therapy against tuberculosis: concept and recent developments. J. Biosci. 48:54. doi: 10.1007/s12038-023-00374-y, PMID: 38088376

[ref48] Wagner D. Young L. S. (2004). Nontuberculous mycobacterial infections: a clinical review. Infection 32, 257–270. doi: 10.1007/s15010-004-4001-4, PMID: 15624889

[ref49] Welin A. Hüsler D. Hilbi H. (2023). “Imaging flow cytometry of Legionella-containing vacuoles in intact and homogenized wild-type and mutant Dictyostelium” in Spectral and imaging cytometry. eds. Barteneva N. S. Vorobjev I. A. (US, New York, NY: Springer), 63–85. 10.1007/978-1-0716-3020-4_4 37074657

