# Supplementary material for: Dictyosteliumdiscoideum–Mycobacterium marinum infection model: a powerful high-throughput screening platform for anti-infective compounds
Source: Front Microbiol. 2025 Jul 4;16:1612354. doi: 10.3389/fmicb.2025.1612354 (PMC12271166; doi:10.3389/fmicb.2025.1612354)
Supplement: Supplementary file 1 [file Data_Sheet_1.docx]

Supplementary Material

## 1. Generation of *M. marinum* stocks

- Glycerol stocks of Mm are generated from a liquid culture by adjusting the culture medium to 20% glycerol and aliquoting the desired amount into tubes.
- The stocks can then directly be stored at -80°C or in liquid nitrogen.

## 2. Generation of *D. discoideum* spore stocks

- Grow Dd in suspension (HL5-C medium, 22°C, shaking at 120 rpm) until a density of 1*107 cells/mL.
- Pellet the cells in SoerensenMC by centrifugation (500 g, 10 min, corresponds to 1500 rpm with the Thermo Scientific™ 75003180 rotor), add back SoerensenMC to repeat the wash to eliminate traces of HL5-C.
- Prepare agar dishes with SoerensenMC and 2% Bacto agar (BD, Difco Bacto Agar).
- Plate 2 mL of cell suspension per agar plate, if necessary, remove excess liquid by pipette after allowing cells to sediment on the agar plate.
- Put lids on and incubate upside down at 22ºC for at least 24h (up to 3 days) in a closed box with humid paper towels to prevent drying out of the plates.
- After fruiting bodies are generated, smash them on the agar by tapping the Petri dish vertically. Then, wash spores from the plate with SoerensenMC. For this, add 1 mL of SoerensenMC, swirl, and collect until most material is removed from the agar dish. Pool the suspension from multiple Petri dishes, if necessary.
- Measure spore density with the Countess (as described above for cell suspensions).
- Wash the spore suspension by centrifuging (500g, 10 min, corresponds to 1500 rpm with the Thermo Scientific™ 75003180 rotor), decanting the supernatant and resuspending in a volume of 10% glycerol in SoerensenMC to adjust the density to ca 107 spores per mL.
- Freeze in aliquots from 0.2 to 1mL and store overnight at -80°C in a Nalgene freezing box with isopropanol before transferring tubes to storage at -80°C or in liquid nitrogen.
- Check quality of the stock by thawing one aliquot and passaging the cells for one week.
- SoerensenMC 10x:
- Dissolve KH2PO4 19.97 mg; Na2HPO4*H2O 3.56 mg; MgCl*6H2O 0.1 mg; CaCl2*6H2O 0.1 mg in 1L double distilled water.
- Filter-sterilize, store at room temperature, dilute 1:10 in double distilled water for SoerensenMC 1x, adjust pH to 6.0 with NaOH or H3PO4.

## 3. *M. marinum* quantification

For calibration of optical density measurement to density of a liquid Mm culture, we used the optical bacterial cell counter Quantom Tx^TM^ (Logos Biosystems). Briefly, we grew bioluminescent or GFP expressing Mm to ODs up to 1.8 prepared serial dilutions and measured the respective OD. Subsequently we prepared the samples according to the manufacturers recommendations to be analyzed with the cell counter. Correlating the results with the previously measured ODs yielded the calibration curve presented and a linear fit resulted in the following formula for the bacterial density: $D=\frac{\left( \left[ \mathrm{OD} \right]- 0.082 \right)}{{3.4309*10}^{-9}}$

Supplementary fig. 1: OD calibration curve. The x-axis shows bacteria per mL as measured by the Quantom Tx^TM^, the y-axis shows optical density of the sample. Each data point represents one measured sample. The curve pools samples from two strains, a strain expressing the bacterial lux-operon and a strain expressing GFP.

**4. Calibration of RFU to *D. discoideum* cell number in the plate reader**

The calibration of bioluminescent Mm to RLU obtained in a plate reader, has already been documented (25, 26). To demonstrate the linear relationship between cell density and RFU measures in a plate reader monitoring of Dd expressing mCherry from the *act5*-locus (45) , cultures were grown in suspension to a high density, performed a serial dilution, measured the samples with the Countess (as described above) in four technical replicates, plated 20 µL of the same sample in four technical replicates into a 384-well plate (Interchim FP-BA8240) and obtained RFU under the same parameters as for our growth assay.

Supplementary fig. 2: RFU calibration curve. The x-axis shows averaged cells per mL as measured by the Countess cell counter, the y-axis shows averaged RFU of the sample. Each data point represents the average over four technical replicates of measurements from both readouts. The error bars are the corresponding standard deviations.

| OD | bacteria/mL |
| --- | --- |
| 0.4 | 9.27E+07 |
| 0.45 | 1.07E+08 |
| 0.5 | 1.22E+08 |
| 0.55 | 1.36E+08 |
| 0.6 | 1.51E+08 |
| 0.65 | 1.66E+08 |
| 0.7 | 1.80E+08 |
| 0.75 | 1.95E+08 |
| 0.8 | 2.09E+08 |
| 0.85 | 2.24E+08 |
| 0.9 | 2.38E+08 |
| 0.95 | 2.53E+08 |
| 1 | 2.68E+08 |
| 1.05 | 2.82E+08 |
| 1.1 | 2.97E+08 |
| 1.15 | 3.11E+08 |
| 1.2 | 3.26E+08 |
| 1.25 | 3.40E+08 |
| 1.3 | 3.55E+08 |
| 1.35 | 3.70E+08 |
| 1.4 | 3.84E+08 |
| 1.45 | 3.99E+08 |
| 1.5 | 4.13E+08 |

**Supplementary table 1:** Correspondence table for the OD-bacterial density calibration curve. The left column shows the interpolated OD equivalents of the interpolated bacterial density in the right column.

Supplementary table 2: Overview over used well plates and their measures. This table gives an overview over well plates, their capacity, reference, provider, shape of their wells, diameter or edge length and resulting bottom surface area per well.

| **Well plate and capacity** | **Reference number and manufacturer** | **Round or square** | **diameter/edge length in mm** | **bottom area in cm^2^** |
| --- | --- | --- | --- | --- |
| 96 WP opaque | Thermo Fischer 136101 | Round | 6.55 | 0.34 |
| 384 WP opaque | Interchim FP-BA8240 | Square | 3.65 | 0.13 |

| **Strain** | **Construct name** | **Selection** | **Addgene #** | **Reference** |
| --- | --- | --- | --- | --- |
| *D. discoideum* mCherry | pDM1514 | Hygromycin 50 µg/mL | 108999 | (45) |
| *M. marinum* LuxCDABE | pMV306hsp | Kanamycin 50 µg/mL | 26155 | (26) |
| *M. marinum* GFP | pMSP12::GFP | Kanamycin 50 µg/mL | 30167 | (42) |

**Supplementary table 3:** Overview over used strains and constructs
